# Stakeholder Perspectives on Early Feasibility Studies for Digital Health Technologies in the European Union: Qualitative Interview Study

**DOI:** 10.2196/77982

**Published:** 2025-10-01

**Authors:** Marlen Peseke, Ilja Michaelis, Ornella Tangila Kayembe, Majella Geraghty, Ali McDonnell, Franco Luigi Zurlo, Zoe Sophie Oftring, Nicolas Martelli, Tom Melvin, Sebastian Kuhn

**Affiliations:** 1 Institute for Digital Medicine, University Hospital Giessen-Marburg, Philipps University Marburg, Baldingerstrasse, Marburg, 35042, Germany, 49 642158625; 2 Pharmacy Department, Hôpital Européen Georges-Pompidou, Assistance Publique – Hôpitaux de Paris, Paris, France; 3 School of Medicine, Trinity College Dublin, Dublin, Ireland; 4 Institute for Clinical Trials, University of Galway, Galway, Ireland; 5 SDA Bocconi School of Management, Università Bocconi, Milan, Italy

**Keywords:** digital health technologies, early feasibility studies, AI-enabled medical devices, regulatory framework, MDR, medical device regulation, qualitative research

## Abstract

**Background:**

Early feasibility studies (EFSs) are small-scale clinical investigations conducted during the early development of medical devices to assess initial safety and performance, especially when bench or in-silico testing is insufficient. While EFSs are well established for hardware devices, their application to digital health technologies (DHTs) including artificial intelligence (AI)–enabled medical devices remains limited. The rapidly evolving regulatory landscape, including the European Union Medical Device Regulation (EU MDR 2017/745) and the phased introduction of the European Union Artificial Intelligence (EU AI) Act, creates additional complexity for DHT developers. Despite the recognized potential of EFSs to support iterative, user-centered innovation, little is known about how European DHT companies and contract research organizations (CROs) perceive and implement EFSs, or what barriers and opportunities exist for broader adoption.

**Objective:**

This study aimed to explore stakeholder perspectives on the use, barriers, and opportunities of EFSs for DHTs in the European Union, and to generate stakeholder-driven recommendations for a harmonized EU-wide EFS framework.

**Methods:**

A qualitative descriptive study was conducted using semistructured interviews with representatives from 12 DHT companies and 3 CROs across a range of company sizes, MDR device risk classes, and clinical domains. Participants were recruited through purposive maximum-variation sampling until saturation was reached to capture diverse experiences in regulatory and clinical evidence generation. Interviews, conducted in November 2024 and January 2025, were transcribed and analyzed using thematic analysis, combining deductive and inductive coding.

**Results:**

Interviews revealed that while EFSs are valued for providing early human-factor feedback and facilitating iterative design improvements, their current use in DHT development is limited. Key barriers include unclear and hardware-centric regulatory requirements under MDR, fragmented and inconsistent interpretations across EU member states, resource and expertise constraints, and limited dialog with regulatory authorities. The anticipated introduction of the EU AI Act is expected to further increase regulatory complexity, with stakeholders expressing uncertainty about overlapping obligations and the risk of slowed innovation. Some companies, particularly larger or AI-focused ones, have proactively prepared for these changes, while others, especially small and medium-sized enterprises, face significant resource challenges. Several companies reported prioritizing the US Food and Drug Administration pathway due to clearer guidance for DHTs and structured timelines. Stakeholders advocated for a harmonized EU EFS program with DHT-specific guidelines, standardized documentation, predictable timelines, and improved communication channels. Several international models were highlighted as best practices.

**Conclusions:**

EFSs remain underused in the EU DHT sector, primarily due to regulatory complexity, fragmentation, and a lack of tailored guidance. A harmonized, DHT-specific EFS framework featuring clearer definitions, standardized processes, and structured dialog between innovators and regulators could accelerate safe, effective, and user-centric digital health innovation. As the MDR and AI Act converge, coordinated regulatory approaches will be critical to balancing innovation, safety, and patient benefit in Europe.

## Introduction

Digital health technologies (DHTs) encompass a broad range of tools that use information and communication technologies to improve health and health system performance [1,2]. These include mobile health apps, telemedicine platforms, wearable sensors, and software for clinical decision support. DHTs may or may not qualify as medical devices, depending on their intended use. When a DHT is intended for purposes such as diagnosis, prevention, monitoring, treatment, or alleviation of disease, it may be regulated as a medical device under relevant jurisdictional frameworks. A specific subset of DHTs, digital therapeutics (DTx) are evidence-based, clinically evaluated software-driven interventions designed to prevent, manage, or treat medical conditions [3].

Terminology and classification vary: in the United States, regulators distinguish between “software as a medical device” (SaMD) and “software in a medical device” (SiMD), whereas the European Union uses the term “medical device software” (MDSW) under Regulation (EU) 2017/745 [4]. Increasingly, DHTs, including DTx, incorporate artificial intelligence (AI), which may trigger additional regulatory requirements under frameworks such as the EU Artificial Intelligence Act (Regulation EU 2024/1689), particularly around transparency, safety, and ethical compliance [5-7].

The unique, software-centered, and iterative life cycle of DHTs poses significant challenges for conventional regulatory and clinical evaluation frameworks [8,9]. As regulatory expectations for robust premarket evidence grow, especially for high-risk technologies, there is an urgent need for flexible and efficient evaluation approaches [6,7,10,11]. A comprehensive, lifecycle-based strategy for clinical evidence generation is essential to ensure timely, safe, and effective integration of DHTs into health systems [12,13].

An emerging strategy to address these challenges is the use of early feasibility studies (EFSs) as part of the development pathway. Historically, EFSs were developed to support the early evaluation of traditional high-risk or implantable medical devices, where in-silico or bench testing was insufficient and real-world human data were needed to guide design. EFSs are small-scale clinical investigations of a product at an early stage of development, typically before the product design has been finalized [14,15]. They assess initial clinical safety and performance when bench or in-silico testing is insufficient. Such studies usually enroll fewer than 15 to 20 participants and evaluate feasibility under real-world conditions. Findings from EFSs inform iterative design improvements and further clinical research strategies.

Although EFSs can be conducted within the current European regulatory framework, Europe lacks a dedicated, structured program comparable to that of the United States [16]. The ongoing Harmonized Approach to Early Feasibility Studies for Medical Devices in the European Union (HEU-EFS) project aims to address this gap by developing and piloting a standardized and widely applicable framework for EFSs. Its goal is to support timely, safe innovation in medical devices (MDs) including DHTs, while aligning with existing EU regulatory requirements and stakeholder needs [17]. The project includes dedicated substudies targeting key stakeholders such as national competent authorities, notified bodies, clinical trial sites, health care providers, and patient organizations, each investigated through tailored research protocols [8,17].

Yet despite this potential, EFSs remain underused in DHT development, reflecting both the absence of tailored guidance and the legacy of hardware-focused regulatory approaches [18,19]. To date, most evidence on EFSs comes from traditional MDs, and little is known about how European DHT companies and contract research organizations (CROs) implement EFSs in practice. This underuse represents a missed opportunity to gather early human-factor and usability data, optimize iterative design, and accelerate safe adoption. No previous research has explored the perceptions, barriers, and opportunities faced by these stakeholders regarding EFSs in the European context, highlighting a critical knowledge gap. This gap is particularly relevant given the current regulatory convergence under the Medical Device Regulation (MDR) and the Artificial Intelligence (AI) Act.

To address this gap, we conducted semistructured interviews with European DHT companies and CROs to explore the current use of EFSs, identify barriers and opportunities, and generate stakeholder-driven recommendations for a harmonized EU-wide framework.

## Methods

### Study Design

We undertook a qualitative descriptive study based on semistructured interviews to explore stakeholder perspectives on the use, barriers, and opportunities of EFSs for DHTs in the EU, and to generate stakeholder-driven recommendations for a harmonized EU-wide EFS framework.

### Participant Recruitment and Sampling

We used purposive maximum-variation sampling to ensure a broad range of perspectives from start-ups, small and medium enterprises (SMEs), large DHT companies, and CROs, representing all MDR risk classes (I–III) and diverse clinical domains. Eligible participants were those directly involved in regulatory or clinical evidence generation for DHTs. Participants were identified through research, personal knowledge, and snowball referrals and were invited via email. A total of 17 potential participants were contacted primarily via email (with an optional follow-up by videoconference or telephone) with 2 not responding. Saturation, defined as the point at which no new subcodes or themes emerged from the interviews, was achieved after the tenth interview, with no new subcodes emerging thereafter. However, 5 additional interviews that had already been scheduled were completed (final sample: n=15) and contributed minor elaborations without altering the overall coding structure.

### Data Collection

We developed a semistructured interview guide (see Multimedia Appendix 1) informed by 2 reviews conducted as part of the HEU-EFS project: (1) a systematic review of the MDR (EU) 2017/745, relevant International Standards Organization (ISO) standards (eg, ISO 14155), and European/international guidance on clinical investigations; and (2) a scoping review of the evidence base for EFSs in DHTs. These reviews, involving partial author overlap with this study, guided the theoretical framework of the interview guide, the thematic structure and question development. The interview guide was piloted internally to ensure clarity and relevance. The guide covered five key domains: (1) understanding the clinical evidence required for Conformité Européenne (CE) marking, (2) experiences with EFSs, (3) clarity and applicability of the EU regulatory framework, (4) the EU AI Act, and (5) expectations for a future EU EFS program.

All interviews were conducted in English via Microsoft Teams by the last author (SK), with 2 additional team members (MP and IM) present to assist with note-taking and technical support. Interviews lasted an average of 48 (range 39‐62) minutes, were video-recorded, and transcribed verbatim using the platform’s built-in transcription software. Transcripts were checked for accuracy against the recordings.

### Data Analysis

A total of 2 researchers (MP and SK) independently analyzed the interview transcripts using thematic analysis, supported by MAXQDA Analytics Pro (version 24.7.0; VERBI Software). The initial coding framework was developed deductively from the interview guide, with additional categories added inductively as new themes emerged. Our analysis was informed by Mayring qualitative content analysis, using predefined categories to structure and interpret the data in a theory-driven manner. Coding discrepancies were discussed and resolved by consensus, involving a third team member (IM) when necessary. The final thematic structure formed the basis for interpreting stakeholder perspectives and developing study recommendations.

Initially, the transcribed text was reviewed, and key passages were highlighted in MAXQDA. These are presented as anchor quotations in the following section. In the next step, main categories were identified through deductive category application based on the interview guide; these correspond to the primary headings in the results section. Relevant text passages were then marked accordingly in each transcript. Further subcodes were assigned under the main categories to enable a more nuanced analysis. These subcodes were also used to tag additional text passages within the individual transcripts in MAXQDA. Text segments addressing multiple themes were coded multiple times, that is, assigned to all relevant categories. The list of main categories, subcategories, and their thematic assignments is provided in Table 1.

**Table 1.** Thematic category system developed through qualitative content analysis of interviews with digital health technology (DHT) stakeholders. The table displays major categories and associated subcategories, along with the number of coded references assigned to each. Data were generated from 15 semistructured interviews conducted with representatives from DHT companies and contract research organizations (CROs) across Europe between November 2024 and January 2025.

Category	Value, n (%)
Introductory questions to understanding the clinical evidence required for Conformité Européenne (CE) marking	184 (28.2)
Company focus and type of DHTs	78 (11.9)
Regulatory challenges with classification	50 (7.7)
Form of regulatory advice	32 (4.9)
Primary goals for clinical investigation	24 (3.7)
Experiences with early feasibility studies and early clinical evidence generation	64 (9.8)
Level of experience	41 (6.3)
Challenges	23 (3.5)
Regulatory requirements and standards	123 (18.8)
Clarity and applicability of MDR^a^, guidance, and ISO^b^ for EFSs^c^	45 (6.9)
Specific regulatory challenges	34 (5.2)
Potential influences on approach to clinical investigations	9 (1.4)
Aspects of iterative development	35 (5.4)
European Union AI^d^ Act	101 (15.5)
Impact	14 (2.1)
Organizational preparedness for AI-specific regulations	27 (4.1)
Perceived challenges	30 (4.6)
Perceived potential benefits	30 (4.6)
Expectations for a future EFS program	155 (23.7)
Features	11 (1.7)
Views on regulatory consultation	35 (5.4)
Organizational aspects (timeline, feedback, and support preferences)	74 (11.3)
International perspective	35 (5.4)
Closing questions	26 (4)
Other feedback	26 (4)

a MDR: Medical Device Regulation.

b ISO: International Organization for Standardization.

c EFS: early feasibility study.

d AI: artificial intelligence.

### Ethical Considerations

The study protocol was approved by the Bocconi University Ethics Committee (approval EA000846; November 11, 2024) and conducted in accordance with the Declaration of Helsinki. All participants provided written informed consent in English, including consent for audio and video recording and details on data management. Data were pseudonymized and stored securely on password-protected servers, with access restricted to the research team, in compliance with the EU General Data Protection Regulation (GDPR). Participants were informed of their right to withdraw at any time, and all efforts were made to ensure confidentiality throughout the research process. No compensation was provided. All participant quotes are presented anonymously, and no identifiable images or multimedia are included in this paper or supplementary files.

## Results

### Overview

A total of 15 individual interviews were conducted using a semistructured interview guide with representatives from 12 DHT companies and 3 CROs. Of the interviewees, 9 were female and 6 were male. Interviews ranged from 39 to 62 minutes (mean 48 min). All MDR risk classes and company sizes were represented (see Table 2). A detailed COREQ checklist for reporting qualitative studies is provided as Checklist 1 [20]. A total of 653 codes were assigned. Their thematic assignments to the main categories and subcategories are provided in Table 1. A detailed codebook is provided as Multimedia Appendix 2.

**Table 2.** Company profiles of participants in the study. Data include company size, Medical Device Regulation (MDR) classification, digital health technology (DHT) characteristics, field of application, and registered country location. Data were derived from 15 semistructured interviews conducted remotely between November 2024 and January 2025 with European representatives from DHT companies and contract research organizations (CROs).

Category	Number
Company size^a^	
Large enterprise: ≥250 employees	7
Medium enterprise: 51‐249 employees	4
Small enterprise: 11‐50 employees	3
Micro enterprise: ≤10 employees	1
Risk classification (MDR)^a^	
I	2
IIa	10
IIb	5
III	3
DHT characteristics^a^	
Artificial intelligence-enabled	8
Patient-facing apps	7
Telemonitoring system or platform and devices	5
Predictive analytics platforms	2
Implantable devices	2
Wearables	1
Imaging tools	1
Field of application^a^	
Orthopedics (including spine)	4
Cardiology	3
Birth control, fertility, and obstetrics	3
Infectious diseases	2
Diabetes mellitus	2
Wound management	2
Pulmonary disease	2
Vascular	1
Medication management	1
Endoscopy	1
Radiology	1
Tinnitus	1
Obesity	1
Countries	
Germany	7
France	2
Switzerland	1
The United States	4
Denmark	1

a Notably, several companies managed multiple products spanning different risk classes, DHT characteristics, and application fields, resulting in a higher product than company count.

### MD Qualification

All interviewed companies confirmed that most of their products qualify as MDs under the EU MDR. Some also developed non-MD health care solutions, requiring different regulatory pathways. Challenges were pronounced for hybrid or emerging technologies, where qualification pathways were less clear. Transitioning from the Medical Device Directive (MDD) to the more stringent MDR introduced additional regulatory hurdles, particularly for newer or unconventional products. While some companies found straightforward pathways for AI-driven applications, others struggled with the lack of specific guidance. Some companies initially considered developing new artificial intelligence–enabled medical devices (AIeMDs) but opted to rather integrate AI features into existing MDs due to uncertainties around early clinical evidence requirements.

### Risk Classification

Determining risk classification was also perceived as challenging. Companies often sought external consultation to ensure accurate classification within the MDR framework. Internal and external risk assessment processes as well as engaging in dialog with notified bodies (NBs) and regulatory experts were vital, though consistent MDR interpretation was often lacking.

### Objectives of Early Clinical Investigations

**Figure 1.**
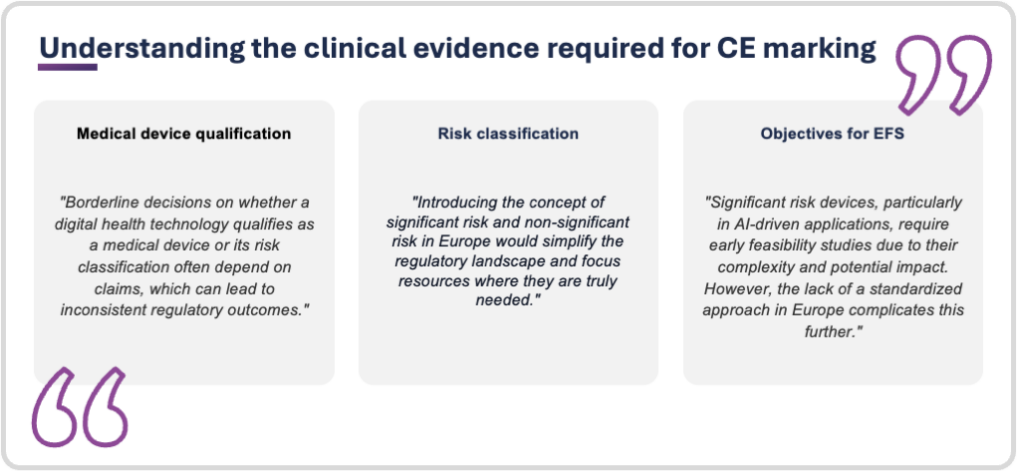
Quotations from interview participants on their understanding of the clinical evidence required for Conformité Européenne (CE) marking of digital health technologies (DHTs). Quotes are grouped into three subthemes: (1) challenges related to medical device qualification and borderline classifications; (2) the role of risk classification in shaping evidence requirements; and (3) objectives and justifications for conducting an early feasibility study (ESF), especially in the context of higher risk classes or artificial intelligence (AI)–driven applications. Quotes are derived from the qualitative interviews with representatives from DHT companies and contract research organizations (CROs) between November 2024 and January 2025.

The primary goals of early clinical investigations (CIs) performed by these companies were multifaceted. Clinical validation of technology was a core objective, with companies aiming to demonstrate the safety and performance of their products in real-world settings. Another significant goal was to gain insights into user experiences, both from clinicians and patients, to refine the technologies. Many companies highlighted the importance of understanding how their DHTs would be used in practice, particularly in terms of clinician and patient workflow. Regulatory milestones, such as achieving CE marking or Food and Drug Administration (FDA) approval, were crucial in guiding these early investigations, with several companies also focusing on provisional reimbursement strategies to support market entry for lower-risk devices. Figure 1 shows the quotations from interview participants on their understanding of the clinical evidence required for CE marking.

### Experiences With EFSs

#### Engagement With EFSs and Decision Drivers

While the concept of EFSs is well recognized, most interviewees indicated that the concept of EFS is not routinely used within the industry. Instead, companies refer to pilot studies, preliminary validation, or early‐stage CIs. Large or AI‐intensive firms were more likely to run multiple EFS or EFS‐like studies annually, driven by regulatory needs, especially for novel AI features or higher-risk classes. In contrast, many smaller DHT companies tried to avoid EFS when possible, due to resource constraints.

#### Key Challenges in Designing and Conducting EFSs

Common challenges mentioned by the interviewees included unclear regulatory expectations for DHTs, limited dialog with regulators, unpredictable timelines, and the lack of specialized DHT expertise. These factors increased costs and operational complexity. Fragmented national requirements and inconsistent regulatory interpretation across EU countries and by NBs further complicated the process. EFSs are described as resource-intensive, particularly burdensome for SMEs with limited budgets and EFS expertise. Companies described varied familiarity of NBs with DHTs and AIeMDs. These challenges collectively deterred many companies from pursuing EFSs as a preferred strategy.

#### Alternative Methods for Evidence Generation

Several companies discussed alternatives to EFSs due to their approach to avoid EFS studies whenever possible. These include conducting simulations with retrospective datasets, which allow for the testing of algorithms or models in a controlled environment without the need for live clinical trials. Others leveraged real-world evidence (RWE) by analyzing patient data from existing databases or partnerships with health care providers. For instance, some companies used synthetic datasets to mimic clinical scenarios, while others relied on feasibility analyses conducted in silico to refine product design and validate initial assumptions. In addition, some participants engaged in pilot collaborations with academic centers, focusing on user feasibility while avoiding formal EFSs. These approaches were favored for their cost-effectiveness, efficiency, and reduced regulatory burden compared to traditional EFSs.

#### The Missed Opportunity of EFSs

**Figure 2.**
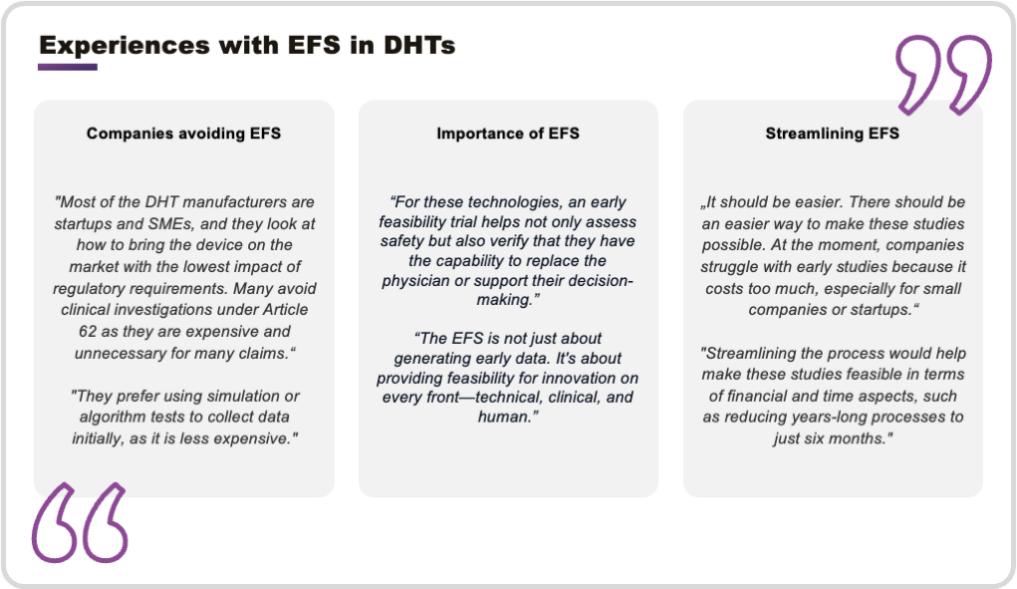
Quotes from digital health technology (DHT) interview partners reflecting their experiences with early feasibility studies (EFSs) in DHTs. Quotes are grouped into three subthemes: (1) reasons some companies avoid EFSs (eg, cost, regulatory burden); (2) perceived importance of EFSs for technical, clinical, and human-centered innovation; and (3) the need to streamline EFSs procedures to improve feasibility for smaller companies. Quotes are derived from the qualitative interviews with representatives from DHT companies and contract research organizations (CROs) between November 2024 and January 2025.

Despite the challenges and alternatives, some companies emphasized that avoiding EFSs entirely may represent a missed opportunity for DHTs and AIeMDs. EFSs can provide crucial insights early in the development process, enabling timely device modifications before large-scale clinical studies are undertaken. It is about providing feasibility for innovation on every front—technical, clinical, and human. In particular, EFSs allow for the investigation of the “human” aspect of DHTs in clinical settings, capturing real-world interactions between users and devices. This enables the collection of critical usability and performance data in real-world settings. This feedback is invaluable for prototype modifications, refining user interfaces, improving usability for both patients and professional users, and addressing unforeseen issues. Companies advocating for EFSs highlighted its role in optimizing device design, ensuring user-centric solutions, and derisking subsequent stages of development. Streamlining the process would help make EFSs more feasible and attractive, encouraging broader adoption and fostering innovation in DHTs. Figure 2 shows the quotations from interview participants reflecting their experiences with EFSs in DHTs.

### Clarity and Applicability of the EU Regulatory Framework

#### Hardware‐Centric Versus Software‐Driven Realities

While many companies acknowledged improvements in transparency with MDR, most agreed the framework and ISO standards remain primarily oriented toward traditional hardware devices, failing to address DHT-specific needs such as iterative software updates or AI model changes. They emphasized that this hardware-centric focus often overlooks the fundamental nature of SaMD and AIeMDs, leading to significant barriers for early clinical evidence generation.

#### Clinical Investigation Planning

Planning CIs for DHTs was identified as a substantial obstacle. Companies report that the MDR’s requirements for detailed preclinical validation and documentation do not align with the exploratory and adaptive approach characteristic of early-stage development in DHTs. MDR’s focus on extensive prevalidation often conflicts with the rapid refinements and protocol adjustments needed for DHTs, stifling the ability to adapt study designs based on interim findings. Interviewees highlighted that the framework’s one-size-fits-all approach to CI is particularly unsuited to the iterative and dynamic processes central to DHT innovation. Companies also reported a lack of guidance on clinical validation of AIeMDs and on AI feature upgrades within existing MDs.

#### Risk Management Frameworks

Risk management frameworks, which are heavily adapted from traditional MDs, were flagged as insufficiently tailored to DHTs. The inclusion of risks specific to software and algorithms often lacks clarity, leaving companies uncertain about the level of depth and validation required. These ambiguities hinder the effective alignment of risk management practices with regulatory expectations.

#### Fragmented Regulatory Landscape

Fragmented regulations across EU member states further complicate compliance. Companies struggle to reconcile disparate national requirements, particularly when managing cross-border patient data. This fragmentation increases compliance costs, delays timelines, and constrains the dynamic, data-driven nature of DHT development.

#### Familiarity With Standards and Regulatory Pathways

The level of familiarity with MDR and ISO standards significantly shapes companies’ regulatory strategies. Companies with robust internal expertise seem to manage EU compliance more effectively, while those with less experience often rely heavily on external guidance. A notable number of companies prioritize the US FDA regulatory pathway due to its perceived clarity, adaptability, and the availability of DHT-specific guidance. Some explicitly follow a “US first, EU second” approach, noting that this trend has reversed what was previously a “EU first” strategy. Several companies used FDA guidance to address EU regulatory processes due to the lack of DHT-specific EU resources, underscoring the need for targeted EU guidance.

#### Lack of Dialogue With Regulatory Bodies

A lack of regular dialog with national competent authorities and NBs early on and throughout the process was frequently cited as a critical issue. Companies emphasized the need for regular, constructive engagement with regulatory authorities to clarify expectations, address uncertainties, and navigate a regulatory framework that was not primarily designed for DHTs. Such dialog is especially important for clarifying pathways for innovative devices, where companies struggle to fit novel features and methodologies into preexisting frameworks. The absence of these interactions often leaves companies operating under assumptions, increasing the risk of regulatory non-compliance or delays. From the companies’ perspective, the interviews particularly emphasized the insufficient expertise of some NBs in relation to DHTs and the associated complex relationships between SaMD and AIeMD.

#### Addressing Iterative Development Within Regulatory Requirements

**Figure 3.**
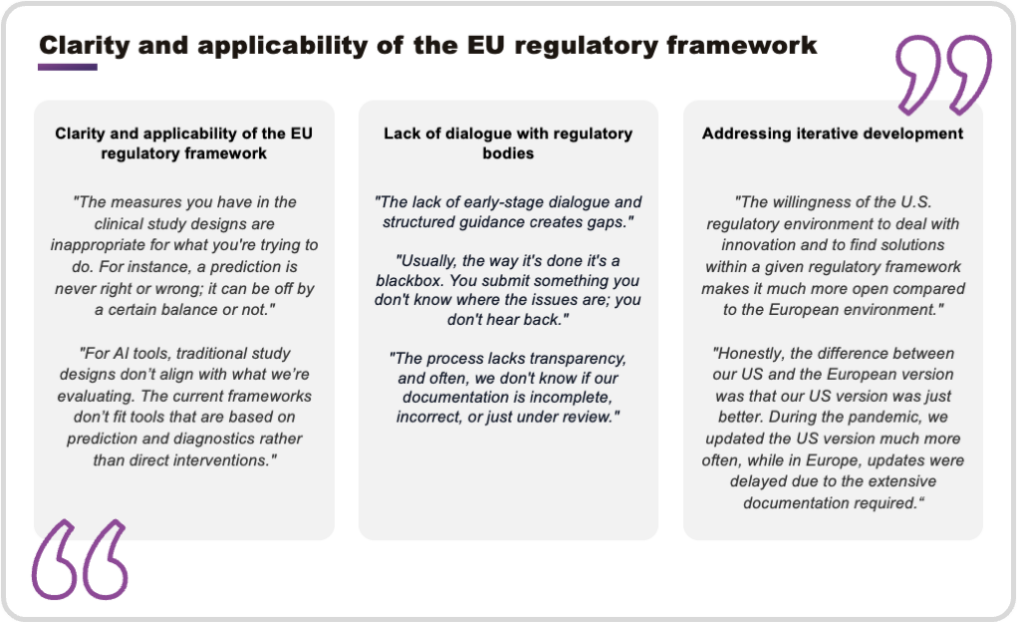
Quotes from digital health technology (DHT) interview partners reflecting their views on regulatory requirements and standards for DHTs in Europe. Quotes are grouped into three subthemes: (1) clarity and applicability of the EU regulatory framework, (2) lack of structured dialog and transparency in interactions with regulatory bodies, and (3) challenges related to iterative product development under European regulatory conditions. Quotes are derived from the qualitative interviews with representatives from DHT companies and contract research organizations (CROs) between November 2024 and January 2025.

The iterative nature of DHT development remains a key regulatory challenge. While companies use strategies such as design change control processes, many still find regulatory timelines incompatible with frequent updates and improvements. This misalignment creates uncertainty about how to manage ongoing changes without triggering new regulatory submissions or extensive documentation. Notably, some companies reported delaying or excluding innovative features from their EU products, prioritizing their introduction in the US market instead. This decision is driven by the perception that the EU regulatory framework’s rigidity, coupled with lengthy review processes and the excessive burden of postmarket clinical follow-up (PMCF) requirements, which necessitate new documentation for each software iteration, limits the ability to efficiently launch updated or enhanced features. Figure 3 shows the quotations from interview participants regarding regulatory requirements and standards for DHTs in Europe.

### EU AI Act

#### Increased Regulatory Requirements for DHTs

The interviews indicate a widespread expectation that the EU AI Act will significantly increase regulatory requirements for DHTs and that these will go beyond the obligations already set out in the MDR. The unclear interplay between the AI Act and MDR has resulted in uncertainty and emphasized the need for clearer guidance. While some stakeholders expressed concerns that this could hinder innovation, especially for smaller enterprises, others identified the AI Act’s staged introduction as an opportunity to progressively clarify regulatory guidance. Transparent enforcement could support a more structured pathway for development, fostering consistency across the EU. Aligning these requirements with international standards would further benefit companies operating globally, reducing regulatory fragmentation and uncertainty.

#### Varied Levels of Preparedness Among Companies

The interviews revealed a significant disparity in how prepared companies are for the EU AI Act. AI-focused companies began preparing years ago, forming dedicated teams to integrate AI-specific standards. In contrast, companies with minimal reliance on AI took a more reactive approach, monitoring regulatory developments without implementing significant changes. Smaller companies expressed concerns about limited resources, potentially putting them at a disadvantage.

#### Potential Benefits of the EU AI Act

Participants identified benefits including enhanced patient safety, greater data transparency, and increased trust in AI-driven healthcare solutions. Clearer AI regulation and an emphasis on accountability and fairness may address bias or misinformation and support public acceptance of these technologies. Some stakeholders noted that a more structured approach could reduce uncertainties in EFSs, providing more predictable pathways to compliance. Alignment with international standards was also seen as crucial for competitiveness.

#### Key Challenges and Risks

**Figure 4.**
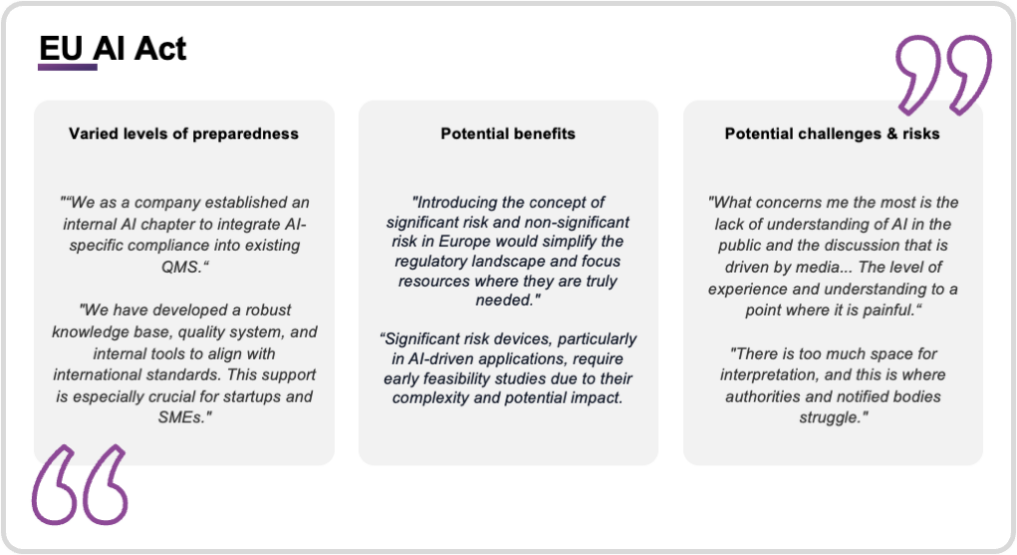
Quotes from digital health technology (DHT) interview partners reflecting their perspectives on the anticipated impact of the EU AI Act. Quotes are grouped into three subthemes: (1) varied levels of organizational preparedness for artificial intelligence (AI)-specific compliance, (2) perceived benefits of the proposed AI risk-classification framework; and (3) anticipated challenges, including interpretative ambiguity and lack of public or institutional understanding. Quotes are derived from the qualitative interviews with representatives from DHT companies and CROs between November 2024 and January 2025.

The increased regulatory complexity is helping to hinder the development of innovative products and services, particularly for small companies. Similarly, society’s noticeable lack of trust in the use of AI emphasizes the need to develop transparent and informative communication measures and establish robust safeguards. Furthermore, the potential for differing interpretations of the AI Act’s provisions across EU member states was flagged as a risk, potentially leading to inconsistent enforcement and added hurdles. Figure 4 shows the quotations from interview participants on their perspectives regarding the anticipated impact of the EU AI Act.

### Expectations for a Future EU EFS Program

#### Key Features of an EU Harmonized EFS Program

Based on the interviews, the following elements were considered critical for an EU harmonized EFS program that also addresses the specific needs of DHTs:

- DHT-specific guidelines
- Harmonization across EU member states
- A clear EFS pathway
- A transparent and collaborative culture

#### Expectations for Timelines, Documentation, and Feedback

Nearly all interviewees stressed the importance of predictable timelines, with about half considering a 6‐8 week feedback window reasonable. Participants also highlighted a distinction between informal and formal decisions: informal guidance was seen as valuable for addressing ambiguities early in the process, while formal regulatory decisions were viewed as necessary milestones to confirm and secure the development pathway. The interviews also underscored the importance of fostering a regulatory culture focused on efficient communication and transparency. Companies called for clear guidance on documentation requirements through standardized templates to reduce administrative complexity and ensure consistency across member states.

#### Structural Supports to Enhance EFS Opportunities

Many respondents advocated for a centralized EU-wide portal for templates, guidelines, and updates. They also suggested educational tools, chatbots, and community forums to create a common knowledge base, and training programs and workshops were proposed to help stakeholders navigate the EFS process effectively.

#### Insights From International Models

**Figure 5.**
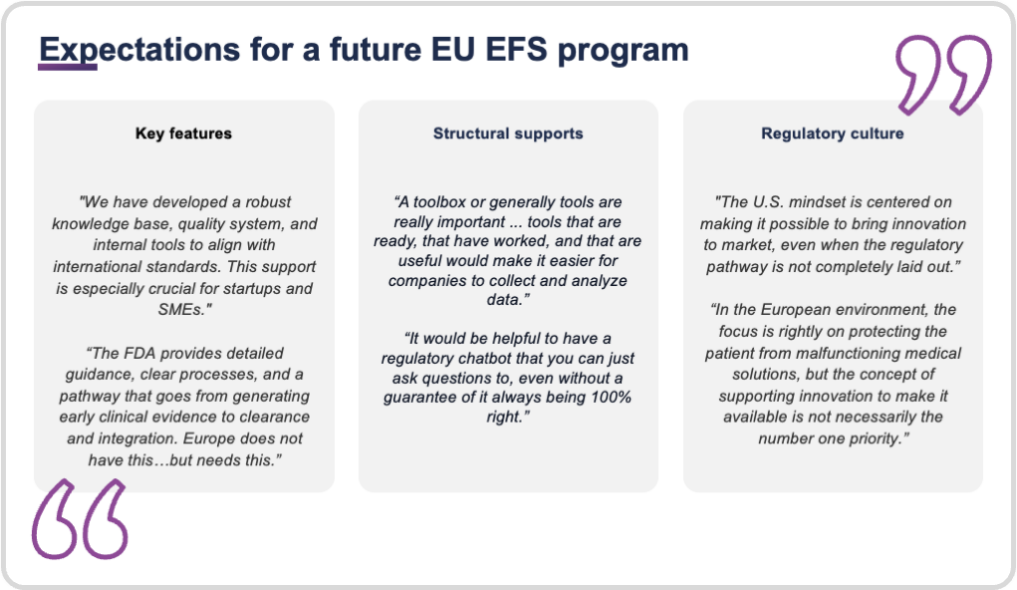
Quotes from digital health technology (DHT) interview partners describing their expectations for the design and implementation of an early feasibility study (EFS) program in the European Union. Quotes are grouped into three subthemes: (1) desired key features, such as structured guidance and small and medium-sized enterprise (SME)–specific support; (2) structural supports, including digital tools and regulatory assistance mechanisms; and (3) perceived cultural differences in regulatory mindset between the European Union and the United States. Quotes are derived from the qualitative interviews with representatives from DHT companies and contract research organizations (CROs) between November 2024 and January 2025.

Several companies pointed to the US FDA’s EFS program as a model for the EU to consider. The FDA’s structured timelines, Q-submission process, and detailed guidance documents were seen as strengths that could be adapted to the EU context. The FDA’s Digital Health Center of Excellence, which provides specialized expertise and support on iterative software updates, AI integration, and data-driven functionalities. Such dedicated support was seen as instrumental in fostering clarity and efficiency for DHT innovators. In addition, the US Predetermined Change Control Plan allows developers to implement iterative software improvements under a predefined regulatory framework, supporting rapid updates of DHTs. As a national experience within the EU, the German Federal Institute for Drugs and Medical Devices DiGA guidance was highlighted as a best practice, particularly for its clarity and specificity in handling digital health reimbursement. Several interviewees suggested that the EU should attempt to harmonize international best practices to foster a globally consistent EFS approach for DHTs and AIeMDs. Figure 5 shows the quotations from interview participants describing their expectations for the design and implementation of an EU EFS program.

## Discussion

### Principal Findings

This study addressed a critical gap in understanding how European digital health companies and CROs perceive and implement EFSs for DHTs and AIeMDs. The principal finding is that EFSs are valued for their potential to generate early safety and performance data, supporting iterative and user-centric innovation. However, their deployment is limited by the perceived complexity of the MDR, fragmented regulatory requirements across EU Member States, and a lack of DHT-specific guidance. These results highlight a persistent tension between the recognized usability of early-stage clinical data and the practical obstacles companies face in generating it.

This finding echoes concerns in the broader literature about the misalignment between conventional evidence frameworks and the iterative, agile development of DHTs [10,13]. Recent frameworks like Evidence DEFINED emphasize the need for proportionate, rapid, and real-world compatible approaches to support early validation [10].

### Underuse of EFSs

Although EFSs are recognized as valuable for providing early safety and performance data—particularly useful for iterative and human-centric innovation—many companies do not routinely deploy them. This underutilization appears driven by several factors: (1) the perceived complexity and resource intensity of conducting formal EFSs under the MDR, (2) the fragmentation of regulatory requirements across EU Member States, and (3) the lack of DHT-specific regulatory guidance. These findings reflect a tension between the acknowledged usability of early-stage clinical data and the current logistical and regulatory barriers that impede their consistent application.

This is consistent with recent literature highlighting that regulatory complexity under MDR—especially due to Rule 11 reclassification—poses substantial barriers to DHT development, disproportionately affecting SMEs [11,13,21]. This has increased documentation requirements and the detail needed for clinical evaluations. The increased complexity and financial burdens disproportionately affect SMEs.

### Fragmented and Hardware-Oriented Regulatory Frameworks

Our results highlight how the EU regulatory framework (notably the MDR) remains largely rooted in the paradigms of hardware-based medical devices. The MDR’s detailed preclinical validation requirements, paired with risk classification that often relies on broad definitions, create challenges for software-driven, rapidly evolving DHTs. Although the MDR has improved transparency relative to MDD, the companies we interviewed often encounter inconsistent interpretations and processes, particularly when handling complex AI features. This gap in structured early evaluation is also apparent in the broader DHT literature. For instance, a recent scoping review found that only 26% of digital health studies targeting people with multiple sclerosis assessed usability formally, and very few incorporated usability findings into iterative design [22]. This disconnect between development and real-world validation is echoed by Pyper et al [23], who found that most DHTs were evaluated in non–real-world settings, with limited consideration of longitudinal patient outcomes or patient-generated data outside clinical encounters. Coupled with divergent national requirements, these factors deter some developers from initiating EFSs, in favor of alternative evidence-generation strategies or US pathways instead of EU-based EFSs. While an EU-wide harmonized EFS program is currently missing, this gap has been identified and is currently being developed and piloted [17].

Early clinical evidence generation is possible in several ways under the MDR. This includes premarket clinical investigations (under Article 62 or 82 of MDR); the deployment of DHTs at a hospital level using “in-house manufacturing” (Article 5 of MDR); or the collection of clinical data to train or tune the algorithm in a way that presents no clinical risk to study subjects. In this case, it can be challenging to determine if this type of data collection is a “clinical investigation” for the purpose of MDR or not. Adaptive trial designs, or the use of umbrella protocols whereby different iterations or combinations of technologies are evaluated, are particularly relevant to DHTs. These types of trials are particularly challenging in the MDR context as there is a significant regulatory burden in preparing multiple applications for “substantial modifications” of the study [24].

As also highlighted in the literature, the lack of harmonized global standards creates inefficiencies, leaving developers to navigate inconsistent requirements across regions [5,25,26]. This gap in alignment complicates the integration of these technologies into health care systems, as existing regulatory mechanisms lack the flexibility to evaluate DHT-specific needs [27]. Jeary et al [28] propose a regulatory framework emphasizing closer collaboration between competent authorities and developers, including mechanisms for identifying acceptable changes, periodic software update reporting, and stakeholder engagement to monitor developments. The framework highlights the importance of partnerships between technology companies and health care entities to facilitate dialog and align innovations with clinical needs. This approach aims to balance technological advancements with robust oversight, ensuring safety and efficacy in digital health care tools. Jiang et al [29] highlight the critical role of notified bodies in assessing the conformity of wearable DHTs to ensure safety, accuracy, and regulatory compliance. Fostering dialog between them is highlighted as pivotal.

### Expectations for the EU AI Act

Companies widely expect the AI Act to add another layer of regulation, potentially compounding the compliance obligations they already face under the MDR. Although larger companies or those deeply immersed in AI have formed dedicated compliance teams and appear relatively well-prepared, smaller organizations express apprehension about resource constraints and the risk of slowed innovation. Nonetheless, many anticipate that a structured AI regulatory framework could help clarify methodological and documentation requirements, potentially leading to more predictable pathways for early clinical research.

All medical devices that incorporate AI could be classified as high-risk under the AI Act, which brings additional requirements [30]. Manufacturers must comply with the requirements of the AI Act (Title II, Chapter 2) and the MDR (safety and performance requirements) for AI-based medical devices simultaneously. This can bring additional challenges, including requirements for risk management systems, technical documentation, quality management systems, and postmarket surveillance. Recent analyses have identified specific misalignments between the AI Act and the MDR in key definitional areas, such as “user,” “provider,” and “risk” that could create regulatory ambiguity unless clarified [31,32]. As highlighted by Gilbert et al [6] and Fraser et al [5], these overlapping frameworks may stall innovation unless harmonized pathways and coordinated oversight mechanisms are established.

### A “US-First, EU-Second” Approach

Several interviewees reported prioritizing the US pathway, due to the availability of specific FDA digital health guidance, established interactions through Q-submissions, and structured timelines for feedback. The FDA’s Early Feasibility Study program, the Digital Health Center of Excellence, and the concept of predetermined change control plans for software updates were viewed as supportive mechanisms for iterative innovation. This trend mirrors previous publications that highlight the FDA’s comparative advantage in digital health through dedicated, DHT-specific regulatory infrastructure [7]. The US model reflects an adaptive governance approach, responsive to the needs of software-based and AI-driven technologies, that could inform European regulatory evolution [5,7]. By contrast, EU developers currently face lengthier and less defined processes, prompting some companies to delay or even forgo certain innovative features in their EU product lines or to change their corporate strategy to first seek authorization in the United States, which has also been reported in literature [33]. The impact of the significant reorganization of the FDA may challenge this dynamic [34].

### Expectations of Companies for a Future EU EFS Program

The interviews highlighted that a future harmonized EU-wide regulatory framework for an Early EFS Program could significantly support the development of novel DHTs. While the FDA’s EFS program does not explicitly exclude DHTs, it was not designed with them in mind and does not explicitly foster them. Companies expect that clear regulatory structures and standardized templates would help decrease existing uncertainties and reduce administrative complexity. Reliable communication processes between innovators and regulatory authorities, early and throughout the process, were considered crucial for building trust and enabling effective collaboration. In addition, low-threshold offerings such as chatbots or community forums could provide an initial means to establish a shared knowledge base and facilitate interaction. Furthermore, the respondents emphasized that existing experiences and programs from other countries—particularly those of the US FDA—could serve as valuable models for designing a European EFS program. However, if such a framework is to succeed, it must avoid replicating hardware-centric assumptions and instead be co-designed with digital health stakeholders to reflect the agile development, software iteration cycles, and risk profiles of DHTs. This user-centered co-creation would help ensure the framework is proportionate, flexible, and aligned with real-world innovation practices [22,23]. Importantly, building public and professional trust in the responsible use of AI-enabled technologies will require not only technical assurance, but also transparency, accountability, and institutional safeguards factors, which recent research shows are more influential than concerns about AI itself [35,36]. Patients and the public are often less distrustful of AI as a technology and more concerned about whether health care organizations can be trusted to implement it ethically and responsibly. The ongoing “Harmonized Approach for Early Feasibility Studies for Medical Devices in the European Union” could provide the evidence base and blueprints for such a harmonized framework [17]. In addition, several of the tools and forums mentioned by stakeholders (eg, educational resources and community platforms) are currently being developed within the HEU-EFS initiative to support stakeholder education and engagement.

### Limitations

First, our sample, while reasonably broad, may not encompass the full range of business models or specialties within digital health. Second, the scope of the interview guide was shaped by our previous knowledge and by the nascent regulatory debates around EFSs and AIeMDs. Some emerging or less common regulatory issues may not have surfaced. Third, we based our analysis on self-reported experiences, which could be influenced by corporate strategy or previous regulatory successes and setbacks. And fourth, as this study focuses exclusively on DHT companies and CROs, the perspectives of other stakeholders, including regulators, health care providers, and patients, are not captured here. However, these are being addressed through other strands of the HEU-EFS research program. Future research could benefit from triangulating interview data with empirical analysis of regulatory documentation and by incorporating insights from complementary stakeholder studies, including those involving national competent authorities, NBs, clinical trial sites, and patient organizations.

### Conclusion

EFSs remain an underused but potentially pivotal tool for supporting safe, agile, and evidence-based innovation in Europe. Our findings suggest that while stakeholders recognize their value, especially for iterative development, their implementation is hindered by regulatory fragmentation, administrative burden, and a lack of DHT-specific guidance under the MDR. As the EU navigates the convergence of the MDR and AI Act, a harmonized, DHT-tailored EU-wide EFS program—drawing on international best practices—could provide the foundation for a more agile and innovation-friendly regulatory environment. This would not only accelerate safe adoption but also strengthen Europe’s position in the global digital health landscape.

## Supplementary material

10.2196/77982 Multimedia Appendix 1 Semistructured interview guide on stakeholder perspectives on early feasibility studies (EFS) for digital health technologies (DHTs) in the European Union.

10.2196/77982 Multimedia Appendix 2 Codebook used for thematic analysis of qualitative interviews. The codebook includes main categories, subcodes, operational definitions, example quotations, and frequency counts.

10.2196/77982 Checklist 1 COREQ checklist.

## Abbreviations

AI Act: Artificial Intelligence Act
AIeMDs: artificial intelligence–enabled medical devices
CE: Conformité Européenne
CI: clinical investigation
CRO: contract research organization
DHT: digital health technology
EFS: early feasibility study
FDA: Food and Drug Administration
GDPR: General Data Protection Regulation
ISO: International Standards Organization
MD: medical device
MDR: Medical Device Regulation
NB: notified body
SaMD: Software as a Medical Device
SiMD: Software in a Medical Device
SME: small and medium enterprise
