# Supplementary material for: Stakeholder Perspectives on Early Feasibility Studies for Digital Health Technologies in the European Union: Qualitative Interview Study
Source: J Med Internet Res. 2025 Oct 1;27:e77982. doi: 10.2196/77982 (PMC12500223; doi:10.2196/77982)
Supplement: Multimedia Appendix 1 [file jmir-v27-e77982-s001.docx]

### Appendix

**Appendix 1: Semi-structured interview guide** **on stakeholder perspectives on EFS for DHTs in the EU**

**1. Introductory Questions to understanding the clinical evidence required for CE marking**

- “Can you share an overview of your company's focus and recent medical devices in digital health technologies?"

Backup Questions:

- "What types of digital health technologies (DHTs) have you/are you currently developing, and what are their intended uses?"
- "What was your experience in deciding if your product qualifies as a medical device (or not) under the current EU regulations?"
- "How did you determine the risk classification of your product, and were there challenges in this process?"
- "Did you seek any regulatory advice to support your clinical development plan? If so, what type and how useful was it?"

"For early clinical investigations, what were the primary goals, such as:

- Clinical validation of the technology
- To understand patient or clinician experiences in using the DHT
- To assess safety/performance/effectiveness outcomes of the DHT
- To achieve CE-marking and provisional reimbursement
- Other

**2. Experiences with Early Feasibility Studies/early clinical evidence generation**

- "Can you describe any recent experiences with early feasibility studies (EFS)/early clinical evidence generation for your digital health products?"

Backup Questions:

- "How frequently do you engage in EFS, and what drives the decision to initiate these studies?"
- "What major challenges have you encountered when designing or conducting EFS for digital health technologies?”
- if no EFS experience: why not?
- If EFS planned but not conducted: Why was it not conducted/cancelled?

**3. Regulatory Requirements and Standards**

- "How clear and applicable do you find the EU regulatory framework, especially MDR guidance and ISO standards, for early clinical investigations in DHTs?"

Backup Questions:

- "Do you feel that the current regulatory guidelines adequately cover EFS for digital health technologies, or are there areas that lack clarity?"
- "Are there specific regulatory aspects (such as risk management or clinical investigation planning), that have been challenging?"
- "How does your familiarity with these standards influence your approach to early clinical investigations?"
- "How do you address the iterative nature of DHT development within the framework of regulatory requirements?"

**4. EU AI Act**

- "How do you anticipate the EU AI Act will impact the regulatory requirements for DHTs, especially concerning early feasibility studies / clinical investigations?"

Backup Questions

- "How is your company preparing to comply with AI-specific regulations in addition to existing medical device standards?"
- "Do you see potential benefits or challenges from the EU AI Act that could influence the way DHTs are developed and assessed in early feasibility stages?"

**5. Expectations for a Future EFS Program**

- "What features or support would be most valuable in a potential EU harmonized EFS program for DHTs?"

Backup Questions:

- "How important is early-stage regulatory consultation, and how could it be improved to support EFS in DHTs?"
- "What are your expectations regarding timelines, documentation, and feedback in an EU EFS program?"
- "Are there specific supports or structures that you believe would strengthen EFS opportunities in the EU?"
- "Are there any international EFS programs, such as the US FDA’s EFS program or the Center for Digital Excellence, that you feel the EU could model to improve its own EFS framework?"

**6. Closing Questions**

- "Do you have any additional feedback or thoughts on Early Feasibility Studies/early clinical evidence generation in DHTs that we haven’t yet discussed?"
