# Supplementary material for: Stakeholder Perspectives on Early Feasibility Studies for Digital Health Technologies in the European Union: Qualitative Interview Study
Source: J Med Internet Res. 2025 Oct 1;27:e77982. doi: 10.2196/77982 (PMC12500223; doi:10.2196/77982)
Supplement: Multimedia Appendix 2 [file jmir-v27-e77982-s002.docx]

| **Main category**  **(n overall statements in this main category)** | **Sub‑code (short label)** | **Operational definition** | **Example quotation** | **n (statements)** |
| --- | --- | --- | --- | --- |
| **1. Introductory questions to understand the clinical evidence required for CE marking**  **(184)** | Company focus/type of DHTs | Code a text segment when the interviewee specifies the medical purpose, target user or clinical application area of the digital‑health technology (DHT) that is under discussion.  Include statements naming indications, patient populations, clinical settings or intended use (diagnosis, monitoring, therapy, decision support, etc.).  Exclude purely corporate matters (e.g., funding, staffing, market size) unless they simultaneously mention the product’s medical purpose. | “We have two modules, the one on the doctor and therapist side and the other one on the patient side... The patient side is only the one where the patient enters the data and gives information about the status and the doctor or therapist module is the one who's the medical device because.” (P09) | 78 |
|  | Regulatory challenges with classification | Code when the interviewee describes uncertainty, disagreement or negotiation regarding risk‑class assignment.  Include references to borderline products or shifts in class during development.  Exclude simple statements of the final risk class if no challenge is mentioned. | “I know that we had quite a lot of discussions with the notified bodies on the risk classifications.... What is the scope of the medical device and the purpose because some of the features that we developed for different medical devices.  We are supposed to be a medical device under the MDR, but the FDA had a different view on that. So we had a lot of discussions back and forth..” (P12). | 50 |
|  | Form of regulatory advice | Code when an organization describes receiving or actively seeking input from external individuals or bodies (e.g., consultants, regulatory specialists, accelerators, legal advisors, or trade associations) to inform their regulatory strategy for clinical investigations.  Include formal paid consultations, structured advisory programs, or informal mentoring that influence regulatory decisions.  Exclude internal brainstorming, in-house compliance team discussions, or hypothetical mentions of advice that was never actually received. | “We had many talks with different stakeholders, external consultants and some legal consultants about this topic and even they didn't know which medical class to apply on that one, because in the past like 2015/16, that was quite a new topic.” (P01) | 32 |
|  | Primary goals for clinical investigation | Code when the interviewee states the purpose or rationale for conducting any early clinical investigation, pilot or EFS  Include goals such as: - clinical validation   - patients’ experiences - safety/performance/effectiveness outcomes - CE-marking and provisional reimbursement - other goals   Exclude procedural comments that do not express a goal | “The primary goal are usually two things… Safety is the first aspect and then efficiency and what efficiency really means is something that's subject to discussion, in particular in our setting.” (P08). | 24 |
| **2. Experiences with Early Feasibility Studies/early clinical evidence generation (64)** | Level of experience | Code when an interviewee quantifies or qualifies prior hands‑on experience with EFS or equivalent pilots (years of activity, number of studies, regions covered).  Include time‑based (“five years”), frequency‑based (“three EFS per year”) or scope‑based (“across two risk classes”).  Exclude mentions of general clinical research experience or studies unrelated to feasibility/evidence generation in early product development phases. | “We can already get some insights from these early feasibility studies.” (P13). | 41 |
|  | Challenges | Code passages describing difficulties specific to planning, conducting, recruiting for or analyzing an EFS.  Include resource constraints, ethics approval, patient recruitment, site logistics, data capture, statistical power.  Exclude challenges confined to interpreting a legal or normative requirement (Those are coded under 3. Regulatory Requirements and Standards) | “Identifying that small, narrow, patient population that benefits the most from your technology that is sometimes challenging.” (P13). | 23 |
| **3. Regulatory requirements and standards (123)** | Clarity and applicability of MDR, guidance and ISO for EFS | Code any evaluation of how clear, complete or suitable the EU MDR, MDCG guidance or ISO standards are for early clinical trials of DHTs.  Include comments on transparency, scope, relevance.  Exclude references to a special challenge (Those are coded under Specific regulatory challenges) | “The guidance, the guidance documents, the information that is available to us is not sufficient to guide us through the process.” (P05). | 45 |
|  | Specific regulatory challenges | Code when the interviewee describes difficulties in implementing or complying with a specific clause or requirement of the MDR (e.g., Annex XIV clinical evaluation plan), MDCG guidance, or ISO standard (e.g., ISO 14155).  Include references to documentation burdens, clinical evidence thresholds, software conformity challenges, or regulatory expectations tied to a specific legal requirement.  Exclude general or abstract concerns about regulatory complexity or clarity, which are better coded under Clarity and applicability of MDR, guidance and ISO for EFS. | “It there should be an easier way to make these studies possible, because at the moment they struggle with early studies with feasibility studies because it costs too much even for the small companies to start-ups etcetera in this field.“ (P09) | 34 |
|  | Potential influences on approach to clinical investigations | Code when a participant describes how existing regulations, standards, or guidance (e.g., MDR, ISO 14155, MDCG documents) have directly affected decisions about study design, endpoints, patient population, or the decision to conduct (or avoid) early feasibility studies (EFS).  Include concrete changes in protocols, adjustments to timelines, or shifts in strategic direction due to regulatory input or guidance interpretation.  Exclude abstract opinions about standards’ clarity without describing an influence | “On that part, we have not so much hands on experience because mostly the academic part takes that part for us.” (P01). | 9 |
|  | Aspects of iterative development | Code when referring to versioning, software updates or other iterative changes during development and their impact on study conduct or documentation.  Include algorithm updates and retention due to iterative development challenges.  Exclude general comments on MDR rigidity without specific mention of iteration. | “Familiar with more modern approaches, and then suddenly you have to go back, so to speak, in their feelings.  And that was quite challenging on how do you keep your development team working, let's say, with an update? (P04) | 35 |
| **4. EU AI Act (101)** | Impact | Code when the interviewee attributes specific observed or projected changes in their company’s workload, development timeline, resourcing, or market access strategy to the requirements or anticipation of the EU AI Act.  Include shifts in release schedules, added compliance burdens, product roadmap alterations, or reprioritization due to classification under the AI Act.  Exclude general opinions about AI regulation that are not tied to organizational impact. | “The AI Act will put every update under high‑risk scrutiny.” (P04) | 14 |
|  | Organizational preparedness for AI-specific regulations | Code statements about internal measures taken (or not taken) to meet AI‑Act requirements (e.g., AI governance boards, QMS updates, SOPs).  Include budget allocation, hiring, training, process redesign.  Exclude abstract opinions about the Act’s difficulty unlinked to concrete actions. | We set up an AI‑compliance squad last year.” (P11) | 27 |
|  | Perceived challenges | Code when a participant describes concrete or anticipated obstacles associated with the implementation or compliance requirements of the EU AI Act.  Include mentions of dual regulatory audits (e.g., MDR + AI Act), increased documentation burdens, concerns over liability, fear of public mistrust, or uncertainty in legal interpretation.  Exclude general AI criticism unrelated to regulation or statements that refer only to MDR or other frameworks without reference to the AI Act. | “Dual MDR‑AI paperwork could overwhelm SMEs.” (P08) | 30 |
|  | Perceived potential benefits | Code when a participant expresses positive expectations about the AI Act’s impact, particularly in relation to healthcare, product development, or the regulatory environment.  Include mentions of improved patient safety, increased patient or public trust, enhanced transparency, harmonized standards across the EU, competitive or market advantage, or regulatory clarity.  Exclude general positive remarks about AI unrelated to AI Act regulation. | “Clearer rules may finally boost payer confidence.” (P13) | 30 |
| **5. Expectations for a future EFS program (155)** | Features | Code when a participant suggests or describes concrete features, tools, or support mechanisms they would like to see in a future EU harmonized EFS program.  Include calls for standardized guidance documents, submission templates, evaluation checklists, digital platforms, best-practice repositories, or shared infrastructures.  Exclude general opinions about EFS programs that do not specify a desired resource, process, or structural feature. | “I think a toolbox or generally tools are really important...In my experiences, collecting data is not the biggest issue, but actually like doing something with the data.  Is extremely time consuming and difficult…Tools that are ready that have worked, that have been evaluated, that are also very useful, that make it easier for companies to collect the data.” (P02) | 11 |
|  | Views on regulatory consultation | Code when the interviewee describes how, when, or under what conditions they prefer to engage with NCAs or NBs for an EFS.  Include preferences for informal vs. formal meetings, Q‑submission analogues, frequency, transparency.  Exclude references to broader regulatory systems, consultation processes outside the EFS context, or general regulatory criticism. | “A clear communication path and an early exchange between the parties would be helpful.” (P12) | 35 |
|  | Organizational aspects (timeline, feedback and support preferences) | Code when an interviewee discusses either experienced or desired procedural aspects of interacting with regulators regarding an EFS, especially timelines, feedback formats, or support mechanisms.  Include specific comments about expected review duration, iterative feedback loops, preferred types of feedback, dedicated regulatory contact person.  Exclude general critiques of bureaucracy or inefficiency of organization. | “Is clarity about timelines and process and steps and it should not be over complicated, especially in these early feasibility studies. (P13) | 74 |
|  | International perspective | Code when the interviewee compares EU practices with non‑EU models (e.g., US FDA EFS or national solutions within Europe or internationally).  Include direct comparisons, references to best practices from other systems, and suggestions to emulate features of non-EU programs.  Exclude general references to international markets or product deployment not linked to EFS processes. | “Further international requirements like FDA clearance or something like that. That really would be very, very helpful.” (P04) | 35 |
| **6. Closing questions (26)** | Other feedback | Code only when the statement provides useful information that does not logically fit any other sub‑code. | “We only have one Ethics Committee for medical devices.” (P014) | 26 |
