# Supplementary material for: Stakeholder Perspectives on Early Feasibility Studies for Digital Health Technologies in the European Union: Qualitative Interview Study
Source: J Med Internet Res. 2025 Oct 1;27:e77982. doi: 10.2196/77982 (PMC12500223; doi:10.2196/77982)
Supplement: Checklist 1 [file jmir-v27-e77982-s003.docx]

**Consolidated criteria for reporting qualitative studies (COREQ): 32-item checklist**

| **No** | **Item** | **Guide questions / description** |
| --- | --- | --- |
| **Domain 1: Research team and reflexivity** | | |
| **Personal Characteristics** | | |
| 1.  Interviewer / facilitator | Which author/s conducted the interview or focus group? | SK conducted all semi structured interviews. MP and IM were facilitators |
| 2.  Credentials | What were the researcher’s credentials? E.g. PhD, MD | SK: Univ.-Prof. Dr. med. et MME  MP: B.A., M.A., doctoral student  IM: LL.B., LL.M., doctoral student |
| 3.  Occupation | What was their occupation at the time of the study? | SK: Professor for Digital Medicine and Director of the Institute for Digital Medicine  MP: Health economist, research associate  IM: Master of Laws, research associate |
| 4.  Gender | Was the researcher male or female? | SK: male  MP: female  IM: male |
| 5.  Experience and training | What experience or training did the researcher(s) have? | SK has > 10 years of experience in qualitative and quantitative research in digital health. MP had been formally trained in qualitative methods including conducting interviews. |
| **Relationships with participants** | | |
| 6.  Relationship established | Was a relationship established prior to study commencement? | Participants were initially contacted via email and received a detailed information and consent form before scheduling interviews. The document outlined the study’s purpose, background, and objectives within the HEU-EFS project explained the voluntary nature of participation, expected duration, confidentiality measures, data protection under GDPR, and researchers' contact information. No prior relationship existed between the researchers and participants before this process. |
| 7. Participant knowledge of the interviewer | What did the participants know about the researcher? e.g. personal goals, reasons for doing the research | The participants were informed about the HEU-EFS project (above) and its aim to develop a harmonized framework for early feasibility studies for medical devices. This was also briefly done at the start of the interview. |
| 8.  Interviewer characteristics | What characteristics were reported about the interviewer/facilitator? e.g. Bias, assumptions, reasons and interests in the research topic | It was reported that the interviewer/facilitators were members of the HEU-EFS project team, with professional backgrounds in digital medicine, clinical research and regulatory science. Each person was shortly introduced with their respective role. |
| **Domain 2 Study Design** | | |
| **Theoretical framework** | | |
| 9.  Methodological orientation and Theory | What methodological orientation was stated to underpin the study? e.g. grounded theory, discourse analysis, ethnography, phenomenology, content analysis | Qualitative content analysis according to Mayring  In preparation, systematic ystematic reviews of regulatory documents (including EU MDR 745/2017, ISO standards, and international guidance) and a formal scoping literature review on early feasibility studies (EFS) in digital health technologies (DHTs) were conducted. Insights from these reviews informed the development of a semi-structured interview guide. |
| 10.  Sampling | How were participants selected? e.g. purposive, convenience, consecutive, snowball | Participants were identified through research, personal knowledge of the HEU-EFS consortium and snowball referrals. |
| 11. Method of approach | How were participants approached? e.g. face-to-face, telephone, mail, email | The participants were invited via email. |
| 12. Sample size | How many participants were in the study? | Fifteen companies were participants |
| 13. Non-participation | How many people refused to participate or dropped out? Reasons? | No participants declined or dropped out. However, two individuals who were contacted did not respond to the invitation email (and one follow-up attempt). |
| **Setting** | | |
| 14.  Setting of data collection | Where was the data collected? e.g. home, clinic, workplace | The interviews were conducted online via videoconferences using Microsoft Teams. |
| 15. Presence of nonparticipants | Was anyone else present besides the participants and researchers? | No, only the participants and the 3 researchers. |
| 16. Description of sample | What are the important characteristics of the sample? e.g. demographic data, date | The sample reflects participants from a diverse range of company sizes, MDR classifications, digital health technologies (e.g., AI-enabled tools, apps, monitoring platforms), therapeutic areas (e.g., orthopedics, cardiology, infectious diseases), and countries of registration. All interviews were conducted between November 2024 and January 2025. A detailed description is given in Table 1. |
| **Data collection** | | |
| 17.  Interview guide | Were questions, prompts, guides provided by the authors? Was it pilot tested? | Yes, the semi-structured interview guide gives a detailed overview of the opening questions and follow-up questions.  Yes, it was pilot tested within the consortium. |
| 18.  Repeat interviews | Were repeat inter views carried out? If yes, how many? | No. |
| 19.  Audio/visual recording | Did the research use audio or visual recording to collect the data? | Yes, all interviews were recorded via Microsoft Teams. |
| 20. Duration | What was the duration of the interviews or focus group? | The interviews lasted an average of 48 minutes (range: 39–62 minutes). |
| 21. Data saturation | Was data saturation discussed? | Yes, saturation was iteratively discussed during the data collection period between the three researchers. Saturation was achieved after the tenth interview, with no new sub-codes emerging thereafter. However, five additional interviews that had already been scheduled were completed (final sample: n = 15) and contributed minor elaborations without altering the overall coding structure. |
| 22. Transcripts returned | Were transcripts returned to participants for comment and/or correction? | Transcripts were not returned to participants. |
| **Domain 3: Analysis and findings** | | |
| **Data analysis** | | |
| 24.  Number of data coders | How many data coders coded the data? | Two researchers (SK and MP) coded the interviews individually and then combined their results. Coding discrepancies were discussed and resolved by consensus, involving a third team member (IM) when necessary. |
| 25. Description of the coding tree | Were themes identified in advance or derived from the data? | The initial coding framework was developed deductively from the interview guide, with additional categories added inductively as new themes emerged during the coding process. |
| 27.  Software | What software, if applicable, was used to manage the data? | Researchers used MAXQDA Analytics Pro (v24.7.0) during the coding of all interviews. |
| 28. Participant checking | Did participants provide feedback on the findings? | No feedback was obtained from participants regarding the results. |
| **Reporting** | | |
| 29.  Quotations presented | Were participant quotations presented to illustrate the themes/ findings? Was each quotation identified? E.g. participant number | Quotations are presented to illustrate the findings in the code book (Appendix 3) with participant number and selected quotes in Figures 1 to 5 without participant numbers. |
| 30. Data and findings consistent | Was there consistency between the data presented and the findings? | Yes, there was consistency between the data presented and the findings. Key themes were supported by illustrative quotes from participants, and findings were clearly derived from the data collected during the interviews. The alignment between reported results and participant input was maintained throughout the analysis and presentation. |
| 31.  Clarity of major themes | Were major themes clearly presented in the findings? | Major themes and sub-themes were clearly present. The results section was organized along these major themes and subthemes (headings and subheadings). |
| 32. Clarity of minor themes | Is there a description of diverse cases or discussion of minor themes? | Topics that were only mentioned by one or few participant(s) were also included in the discussion. |
